# A Conceptual Model to Strengthen Integrated Management of HIV and NCDs among NIMART-Trained Nurses in Limpopo Province, South Africa

**DOI:** 10.3390/clinpract13020037

**Published:** 2023-03-09

**Authors:** Nthuseni Sharon Murudi-Manganye, Lufuno Makhado, Leepile Alfred Sehularo

**Affiliations:** 1 Department of Public Health, School of Health Sciences, University of Venda, Thohoyandou 0950, South Africa; 2 School of Nursing Science, Faculty of Health Sciences, North-West University, Potchefstroom 2531, South Africa

**Keywords:** conceptual model, HIV, NCDs, NIMART nurses, adult primary care guidelines, PHC

## Abstract

Integrated management of human immune deficiency virus (HIV) and non-communicable diseases (NCDs) in primary health care facilities remains a challenge. Despite research that has been conducted in South Africa, it is evident that in Limpopo Province there are slits in the implementation thereof. There is a need to develop a conceptual model to guide in strengthening the clinical competence of nurse-initiated management of antiretroviral therapy (NIMART)-trained nurses to implement the integrated management of HIV and NCDs to improve clinical outcomes of patients with the dual burden of diseases in Limpopo Province, South Africa. This study aimed to develop a conceptual model to strengthen the implementation of integrated management of HIV and NCDs amongst NIMART nurses to improve clinical outcomes of patients with the dual burden of communicable and non-communicable diseases in Limpopo Province, South Africa. An explanatory, sequential, mixed-methods research design was followed. Data were collected from patient records and the skills audit of 25 Primary Health Care (PHC) facilities and from 28 NIMART trained nurses. Donabedian’s structure process outcome model and Miller’s pyramid of clinical competence provided a foundation in the development of the conceptual model. The study revealed a need to develop a conceptual model to strengthen the implementation of integrated HIV and NCDs implementation in PHC, as evidenced by differences in the management of HIV and NCDs. Conclusion: The study findings were conceptualised to describe and develop a model needed to strengthen the implementation of integrated management of HIV and NCDs amongst NIMART nurses working in PHC facilities. The study was limited to Limpopo Province; the model must be implemented in conjunction with the available frameworks to achieve better clinical outcomes.

## 1. Background

Many low- and middle-income countries are still faced with a dual burden of HIV and NCDs despite the great achievement of control of HIV and NCDs over the last decade. Amongst others, the human immunodeficiency virus (HIV) and other nutritional diseases such as hypertension and diabetes continue to account for high mortality rates in low- and middle-income countries. The World Health Organisation (WHO) estimates that more than 75% of people living with HIV on antiretroviral therapy (ART) are susceptible to NCDs due to stimulation of inflammatory markers, adverse event of some ART medicine, tobacco use, and alcohol use. Furthermore, 71% of all deaths globally are due to NCDs [1,2,3]. The prevalence of HIV and NCDs varies from country to country, although we have seen a decline in HIV prevalence in the last decade and an increase in the number of patients accessing antiretroviral therapy due to the large funding that is directed towards HIV programmes as compared to the funding allocated towards NCD programmes [4,5,6]. South Africa has the largest number of PLWH with more than 80% of the people on ART [2]. Despite the progress made in the management of HIV, there is a huge number of HIV patients who develop NCDs or were diagnosed with dual conditions at the start of ART or the start of NCDs treatment [7,8]. Furthermore, most developing African countries have many HIV patients who are affected with NCDs [9].

In South Africa, we have witnessed the successful implementation of the HIV programme through different policy strategies such as 90–90–90. In addition, the mortality rate was reduced by one third due to the implementation of NIMART in PHC facilities. The training of NIMART was led by the South African Department of Health supported by CDC and USAID [4,10,11]. Most professional nurses in South Africa were trained in NIMART following the support (financial and technical support) received from PEPFAR [4]. Despite the NIMART training, the quality of patient care related to NCDs was not a priority until the introduction of the Adult Primary Care (APC) guidelines, which included NCDs treatment guidelines [4,12,13]. The concept of integrated management of HIV and NCDs was realised during the implementation of the ideal clinic strategy, which was piloted in three South African provinces—namely Mpumalanga, Western Cape, and KwaZulu-Natal—in the year 2014. In addition, the ideal clinic strategy developed components and subcomponents that guided the rollout, including integrated management of HIV and NCDs [14,15].

Limpopo province is one of the rural provinces in South Africa, with Vhembe District implementing the integrated management of HIV and NCDs without the support of donor-funded organisations after PEPFAR shifted its support to the 27 districts of the 54 districts in South Africa [16,17]. The health care system in Limpopo Province continues to experience challenges that have serious effects on the implementation of integrated management of HIV and NCDs, including patient clinical outcomes. The literature reviewed established the weaknesses and threats which impact on poor implementation of integrated management of HIV and NCDs. In addition, the study verified the prospects to sustain the quality of the implementation thereof [18,19,20,21].

The implementation of integrated management of HIV and NCDs is the second component of the ideal clinic realisation and maintenance framework. Furthermore, it comprises subcomponents such as clinical supportive management and strengthening of support system, amongst others [14,15,16].

Even though the majority of the professional nurses are trained in NIMART, the APC training is still lagging behind, hence the poor implementation of APC guidelines. A literature review found no model that could be used to strengthen the clinical competence of NIMART nurses to implement integrated management of HIV and NCDs with confidence. Therefore, in this study, the researcher strove to develop and describe a conceptual model which may strengthen the implementation of integrated management of HIV and NCDs amongst NIMART-trained nurses to improve clinical outcomes of patients faced with the dual burden of diseases in Limpopo Province, South Africa. The model is designed to guide clinical competence and implementation of the programme thereof.

## 2. Method

A mixed-methods approach using an explanatory, sequential, mixed-methods design was chosen to conduct a comprehensive literature review, to obtain a descriptive SWOT analysis, and to obtain an in-depth understanding of the views and experiences of NIMART-trained nurses regarding the implementation of integrated management of HIV and NCDs to generate valid conclusions. The study sought to assess the impact of and barriers influencing NIMART training and implementation to produce more complete and well-validated conclusions [22]. The study was conducted in four phases up until the development of the conceptual model. This included a comprehensive literature review, A SWOT analysis, and an exploratory qualitative study.

The conceptual model was developed based on the findings of the first three phases. Donabedian’s (1966) SPO model [22] and Miller’s pyramid of Clinical competence [23] were used concurrently to categorise the characteristics and activities of NIMART nurses’ competencies and the implementation of the integrated management of HIV and NCDs within the PHC context. There is an interdependent relationship between Donabedian’s SPO model and Miller’s pyramid of clinical competence. We used the two frameworks as the starting point for the development of the conceptual model.

Furthermore, a selection of the most appropriate information best describing the phenomenon and the activities necessary for the implementation of integrated management of HIV and NCDs was conducted [24,25]. Table 1 depicts the refinement of the methodology to eliminate overlapping activities.

## 3. Results

NIMART training enables the professional nurses to initiate ART for all patients diagnosed with HIV, while APC training enables the professional nurses to diagnose and manage the patients with NCDs or with both HIV and NCDs. The two training components apply the same principles for learning and acquiring clinical competencies. The findings confirmed that all nurses were trained in NIMART, and only a few of them were trained in APC guidelines. Furthermore, as indicated, only a few were trained in a post-graduate diploma in Primary Health care, with a very small number trained in PALSA plus guidelines. PHC diploma and Palsa Plus entails training in NCDs management [25].

Furthermore, the qualitative results confirmed that professional nurses had confidence in managing HIV as compared to managing NCDs [25]. Such feelings of non-confidence in managing NCDs in a PHC facility contribute to under-diagnosis of NCDs in patients who are HIV-positive, resulting in serious complications including deaths. The study further verified that, as required by the guidelines, patients were not screened for diabetes and at least half of the patients had their blood pressure measured each time they visited a PHC facility, despite the availability of clinical guidelines in PHC facilities. The APC guidelines 2016/2017 regulates the management of adults with communicable diseases (HIV) and non-communicable diseases (hypertension and diabetes), amongst others [25].

The latter was found to relate to the qualitative study results, where it clearly showed that there is an imbalance in HIV and NCDs training because NIMART nurses were not confident or rather competent in managing NCDs. In addition, some nurses felt that NCD management is a medical doctor’s responsibility. Unavailability of the updated APC guidelines in PHC facilities and shortage of medical equipment in some facilities may have also impacted the implementation of integrated management of HIV and NCDs. Both the quantitative and qualitative studies revealed that the PHC facilities do not have all the required clinical stationery for recording the patient care rendered to track the patients’ clinical outcomes over time. Furthermore, nurses become frustrated when there are no registers or patient files. Unclear role clarification compromises the clinical competence of the nurses as the nurses sometimes act as clerks as there is a shortage of administrative staff in PHC facilities.

Besides the imbalance in the HIV and NCDs training that have been discussed, the study further verified the challenges which can impact the successful implementation of integrated management of HIV and NCDs.

There is a vast difference in the implementation of integrated management of HIV and NCDs. Newly trained nurses come into practice without proper training in APC, hence, some patients are not managed in the spirit envisaged in the set guidelines. Usually, the patients are just given HIV treatment without being properly screened for NCDs, or the nurses feel that diagnosing NCDs is a medical doctor’s responsibility. The poor or little support from the NCDs programme managers also contributes to NIMART’s hindrance to providing comprehensive integrated management of HIV and NCDs. The study further identified the use of outdated APC guidelines, thus resulting in poor clinical patient outcomes. In addition, poor infrastructure and poor medical supplies were identified as challenges in this study. The study further confirmed that APC is not considered a requirement for a professional nurse to practice in a PHC setting, and this impacts the quality of patient care rendered [26].

Some opportunities can be strengthened to sustain the implementation of HIV and NCDs. The qualitative study revealed that patient satisfaction is a key to programme implementation, as the care provided becomes patient-centred and stigma is reduced. In addition, support from all stakeholders can change the current picture of integrated management of HIV and NCDs [25]. The study further verified that even if there are challenges, there are suggestions to improve the implementation of HIV and NCDs. This is evident from the qualitative study, which revealed that continuous professional development where professional nurses need to be trained and study further is necessary to improve their skills in providing integrated management of HIV and NCDs. In addition, comprehensive literature review and the quantitative study shows that professional nurses who are already trained in APC can be utilised to teach their peers in PHC facilities [27].

### 3.1. A Conceptual Model to Strengthen the Implementation of Integrated Management of HIV and NCDs

The conceptual model was developed based on the Millers pyramid of clinical competence (1990) and Donabedian SPO model (1966). Miller’s pyramid enabled the researchers to identify the key elements attached to building clinical competence, whereas the Donabedian SPO model allowed the researchers to incorporate the results of all the study phases to inform the conceptual model. The following segment offers a description of the conceptual model.

### 3.2. Miller’s Pyramid

**Figure 1:**
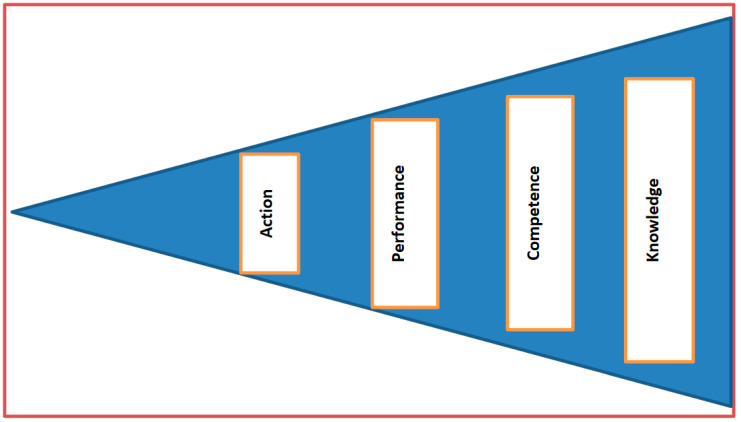
Miller’s pyramid of clinical competence (1990).

Miller’s pyramid describes four levels that rank the clinical competence of the learners in the workplace, as illustrated in Figure 1 Furthermore, it describes the different levels the learners should go through to achieve and be assessed in the programme being delivered [22]. Miller further argued that at the end of any learning programme, the interest is in observing what learners can do to achieve higher levels of professional authenticity. In addition, the type of assessments should be valid to assist the learner to contribute to the improvement of patients’ clinical outcomes. In this study, the workplace is referred to as a district or the PHC facility—while the learners are referred to as NIMART nurses.

### 3.3. Miller’s Pyramid Application

#### 3.3.1. A Person to Have Knowledge on the Implementation of Integrated Management of HIV and NCDs (Knows) Knowledge

According to Miller (1990) [23], a novice must grow into an expert. For the conceptual model, the NIMART nurse must be trained and assessed on the implementation of integrated management of HIV and NCDs to transit from a novice state to an expert state.

A person must have undergone the assessment questions prescribed in the course curriculum. Furthermore, the knowledge that a person has may bring the knowledge attained elsewhere through hearing about it or knowing about it; for instance, a professional nurse who has gathered the facts or has knowledge in the implementation of a certain programme through informal learning. This concurs with Benner’s Novice to Expert Nursing Theory [24], which states that educating nurses is a foundation contributing to the development of specific nursing skills related to clinical guidelines. In this study, the professional nurses are the implementers of integrated management of HIV and NCDs at the PHC level. Therefore, they must know about relevant topics, the stepwise guidelines for adult primary care, and should be assessed on this acquired new knowledge. Missing this first level of competence invariably leads to the poor implementation of integrated management of HIV and NCDs.

#### 3.3.2. Application of Knowledge by NIMART Nurses (Knows How/Understand/Competence)

According to Miller (1990) [23], the professional nurse must be competent enough to apply what they have learnt through case presentation or answering the set questions to demonstrate how much they know of the integrated management of HIV and NCDs and how this is rendered to improve clinical patient outcomes. In other words, NIMART nurses should be able to utilise the knowledge acquired. The knowledge acquired is referred to here as NIMART and APC guidelines. Another study suggests that this level in Miller’s pyramid limits the nurses critical thinking, as nurses merely focus on the set guidelines rather than bringing their own thoughts to the patient care field [25]. However, this suggestion may be challenged, as nurses can only bring their thinking around patient management through thorough and evidence-based documented research.

#### 3.3.3. Demonstration of Learning by NIMART Nurses (Shows How/Performance)

Through the learnt skills and topics, NIMART nurses should be allowed to demonstrate these new skills through simulations and objective structured clinical examinations (OSCEs). This level calls upon NIMART nurses to demonstrate the appropriate skills needed for integrated management of HIV and NCDs. For instance, NIMART nurses should be able to develop and implement a treatment plan for patients diagnosed with HIV and NCDs. Furthermore, they should be able to offer health education appropriate to the diagnosis of the patient. The latter has been documented in some studies where it was confirmed that the value of demonstration in the preparation of nurses has a positive impact on the performance of nurses in the clinical environment [28,29].

#### 3.3.4. Actions by NIMART Nurses (Does/Action)

According to Miller, this level of the pyramid requires the NIMART nurses to perform patient care through all-learnt patient care approaches. This includes the provision of routine patient care to track the clinical outcomes of the patients. Moreover, recording in the patient clinical stationery adds to the actions of the NIMART nurse as such recording provides a baseline for evaluating the care rendered to the patient. During the quantitative study, we found that patients were not screened for NCDs.

Over and above the four levels in the pyramid, Miller affirmed that cognition and behaviour should be displayed by clinicians as an indicator of the nurse being clinically competent. Cognition refers to professional nurses who have never been trained in APC. Anyone who has never been exposed to APC training is certainly more likely to perform poorly compared to those who have received such training. The second term is behaviour, where the NIMART nurses must be tested to see if they can apply what they have learnt into practice. Miller further argued that knowing how does not mean that they will do it on a daily basis. This essentially means that the district is obliged to encourage and support the NIMART nurses who have undergone the APC training to implement integrated management of HIV and NCDs to continuously improve clinical outcomes of the patients. In addition to Miller’s thinking about behaviour and attitude, another researcher reiterates that medical professions, including nursing, depend on individual skills and expertise and rely more on good behaviour and appropriate attitude [28]. Miller further indicates that attitude is the key to clinical competence. In this case, NIMART nurses should demonstrate a willing attitude to transit from a novice state to an expert state in the provision of quality integrated management of HIV and NCDs at the PHC level.

### 3.4. Donabedian SPO Model

**Figure 2:**
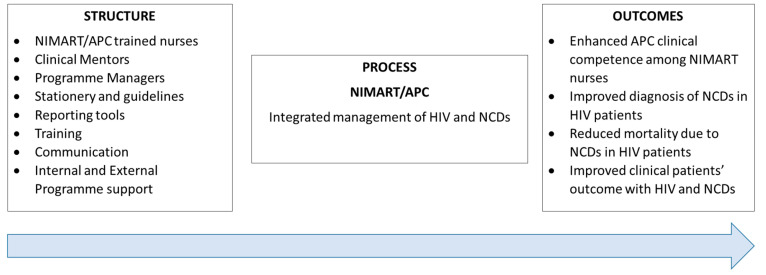
Donabedian SPO model (1966).

According to Donabedian, three elements must be taken into consideration if the quality of patient care must be improved. Donabedian was convinced of three elements, as illustrated in Figure 2.

Application of Donabedian SPO Model to the Findings.

#### 3.4.1. Structure

As described above, the structure in this study refers to the district, the PHC facilities, and the first level of Miller’s pyramid. Following Donabedian’s structure description, the PHC facilities’ hours of operation and type, NIMART/APC-trained nurses, clinical mentors, programme managers, availability of policies and guidelines, availability of clinical stationery, availability of APC guidelines, ART consolidated guidelines, and integrated reporting tools are essential for the implementation of integrated management of HIV and NCDs. In addition to the structure, training, communication, and internal and external programme support may strengthen the implementation of integrated management of HIV and NCDs. This study confirmed that the above-mentioned characteristics are essential in the implementation of integrated management of HIV and NCDs. In addition, NIMART nurses must acquire knowledge to enable them to offer quality care to patients with the dual burden of HIV and NCDs, as indicated in Figure 2.

#### 3.4.2. Process

According to the Donabedian SPO model, all aspects which are considered as the basis of qualified health care should be made available to make sure that patients’ needs are met. In this case, the process is achieved by ensuring that NIMART nurses are assessed on clinical competence. Furthermore, there is an assessment that NIMART nurses should undergo to enable them to diagnose and treat patients with HIV and NCDs. In this study, it was verified that training, programme support, clinical stationery, medical equipment, laboratory equipment, and reporting tools facilitate the process in terms of the implementation of integrated management of HIV and NCDs in PHC facilities. In addition, there should be a concerted use of external or internal motivators to encourage NIMART nurses to adhere to the newly learnt skill.

#### 3.4.3. Outcomes

Donabedian developed a model aimed to assess the quality of care in clinical practice [22]. In this study, the outcomes were achieved by evaluating the screening of diabetes and HPT, for instance if the blood pressure, blood glucose, and urine tests were conducted at first visit [25]. It is evident in this study that there is no adherence to APC guidelines, even though some patients were treated according to guidelines. Furthermore, adherence to APC guidelines may improve mortality rates amongst HIV-infected and NCDs-diagnosed patients.

### 3.5. Application of Miller’s Pyramid of Clinical Competence and Donabedian’s SPO Model to the Conceptual Model

Although Donabedian’s SPO model was designed to evaluate the healthcare system and Miller’s pyramid was developed to assess the doctor’s clinical competence, these two frameworks fit perfectly in the effort to develop a conceptual model to strengthen programme implementation such as integrated management of HIV and NCDs. The two frameworks were merged to forge the conceptual model developed in this study.

**Figure 3:**
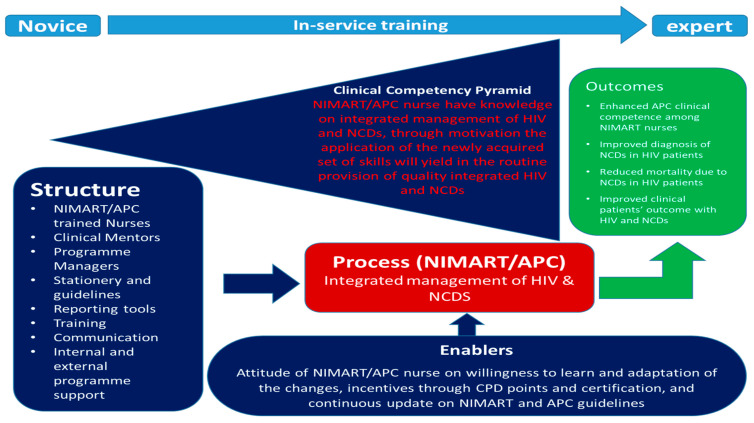
Conceptual model to strengthen the implementation of integrated management of HIV and NCDs among NIMART nurses.

Figure 3 illustrates the conceptual model to strengthen the implementation of integrated management of HIV and NCDs among NIMART nurses in PHC. There is a link between clinical competence, structure, process, and outcomes. Training, assessment, and completion of a portfolio of evidence should not be overlooked or bypassed. Strengthening the implementation of integrated management of HIV and NCDs among NIMART nurses working at PHC facilities purely relies on enhancing clinical competence. According to the study results, the levels of clinical competence are strengthened through the structure and the process within a district. Nurses should be placed in facilities that are flexible for in-service training or continuous professional development. Resources such as policies, guidelines, clinical records, medical equipment, and laboratory equipment must always be available for NIMART nurses to achieve all the levels required for clinical competence.

The process, on the other hand, allows NIMART nurses to clearly define their roles, which subsequently encourages them to develop an interest in enhancing their skills as they will feel a sense of being recognised. In addition, during the process, a supportive environment from the programme managers and external support motivates NIMART nurses to perform much better to improve patient clinical outcomes. The model recognises the NIMART nurse who is not yet trained on APC is placed in a facility where they are expected to implement integrated management of HIV. Often, the NIMART nurses implement the APC without being trained, yet the model emphasizes that novice or untrained nurses should be placed in PHC facilities with necessary skills, or they should be placed in facilities where training can be arranged while they are in service. Structural factors such as availability of medication and clinical records should be a basis for nurses to implement integrated management of HIV and NCDs. For enhanced clinical competence, there should be a process that includes support from various stakeholders, including external motivators. If the structure, process, and clinical competence are addressed, ultimately there will be outcomes such as improved patients’ outcomes.

## 4. Discussion

The purpose of this study was to synthesise the study findings so that we could describe and develop a conceptual model that could guide to strengthen the implementation of integrated management of HIV and NCDs among NIMART-trained professional nurses in Limpopo Province, South Africa. The conceptual model was developed based on Miller’s (1990) pyramid of clinical competence and Donabedian’s SPO model (1966). The study found that the majority of the professional nurses are trained in NIMART, and HIV treatment guidelines are followed by NIMART trained nurses; however, patients who are on ART are not screened for NCDs, as prescribed in the APC guidelines. In addition, the study found that most of the PHC facilities did not have the latest APC guidelines (APC 2016/2017). Furthermore, there is an imbalance in the management of patients with HIV and those with NCDs, and these may be due to the lack of training of NIMART nurses on APC guidelines. The study findings established that essential diagnostic equipment such as a Blood Pressure (BP) machine and a glucometer were available, however, not all patients were screened for BP or glucose. According to Mboweni et al. [27], professional nurses are frustrated when they do not know what to do with patients, especially when they are not trained. Furthermore, another study confirmed that there is little training targeted for professional nurses who are working at the PHC level as compared to the nurses allocated in a hospital setting [28]. The use of developed guidelines to manage both HIV and NCDs enables quality care to patients with comorbidity, therefore, training and mentoring of nurses providing integrated management of HIV and NCDs in rural areas is essential [29,30]. Kane et al. [31] indicated that the availability of diagnostic tools and standardised protocols for disease management in PHC facilities are the key to improving patients’ clinical outcomes.

Poor implementation of integrated management of HIV and NCDs exposes patients to develop complications related to ART side effects or even death. Furthermore, the longer the patient is on ART medication, the higher the risk of the patient developing NCDs [32,33,34]. The estimated number of NCDs-related deaths by 2025 would be prevented if NCDs management in PHC facilities is considered equally important as HIV management; otherwise, the NCDs may compromise the success of the HIV programme [35,36,37,38,39,40,41,42]. It is evident from this study that the clinical competence of NIMART nurses to manage NCDs is low because most of them did not receive the required APC training. There are also other operational factors which are hindering the implementation of integrated management of HIV and NCDs [43,44,45,46]. NIMART-trained nurses did not comply with the APC guidelines, which compromised integrated management of HIV and NCDs including the quality of patient care. The latter deemed it necessary to develop a conceptual model to strengthen the implementation of integrated management of HIV and NCDs in Limpopo Province.

## 5. Conclusions

The implementation of integrated management of HIV and NCDs has proven to increase patient clinical outcomes since its adoption in the last decade. Therefore, training of NIMART nurses on the updated APC guidelines is essential. Enhancing clinical competence among NIMART nurses in Limpopo through training, support from programme managers, availability of equipment and medication, and supporting continued NIMART professional development can assist in the improvement of integrated management of HIV and NCDs at the PHC level in Limpopo Province.

However, the implementation of integrated management of HIV and NCDs by NIMART-trained nurses is still a challenge. It is evident from the study findings that many factors such as clinical competence and health care systems influence how NIMART nurses implement the APC guidelines. The developed conceptual model, therefore, has the aptitude to strengthen the implementation of integrated management of HIV and HIV, thus improving patient clinical outcomes.

## 6. Limitations of the Study

This study was limited to one district in Limpopo Province. Moreover, the focus was on PHC facilities. However, the findings are significant to other rural provinces in South Africa.

## 7. Practical Implications of the Study

The developed conceptual model could assist in strengthening the clinical competence of NIMART-trained nurses, including the proper implementation of integrated management of HIV and NCDs in PHC facilities. Moreover, the conceptual model may be used as a reference during the development of clinical guidelines to ensure that clinical competence is not overlooked.

## Figures and Tables

**Table 1:** QUAN–QAUL methodology.

Phase	Design	Population	Sampling	Sampling Size	Context
Phase 1	Comprehensive literature review	Policies, guidelines, and full text articles	Purposive sampling	16	South African documents for comprehensive literature review
Phase 2	SWOT analysis	Facility data, skills audit patients’ files	stratified simple random sampling	25	
Phase 3	Exploratory study	NIMART Trained Nurses	Purposive sampling	28	All Vhembe district PHC/CHC facilities for SWOT analysis and exploratory study
Phase 4	Development of Conceptual model	PHC facilities	-	-	-
		NIMART Trained nurses			

## Data Availability

On request from the authors.
